# Supplementary material for: Evolutionary Diversity of Bat Rabies Virus in São Paulo State, Brazil
Source: Viruses. 2025 Jul 30;17(8):1063. doi: 10.3390/v17081063 (PMC12390695; doi:10.3390/v17081063)
Supplement: Supplementary file 1 [file viruses-17-01063-s001.zip › viruses-3611479-supplementary.pdf]

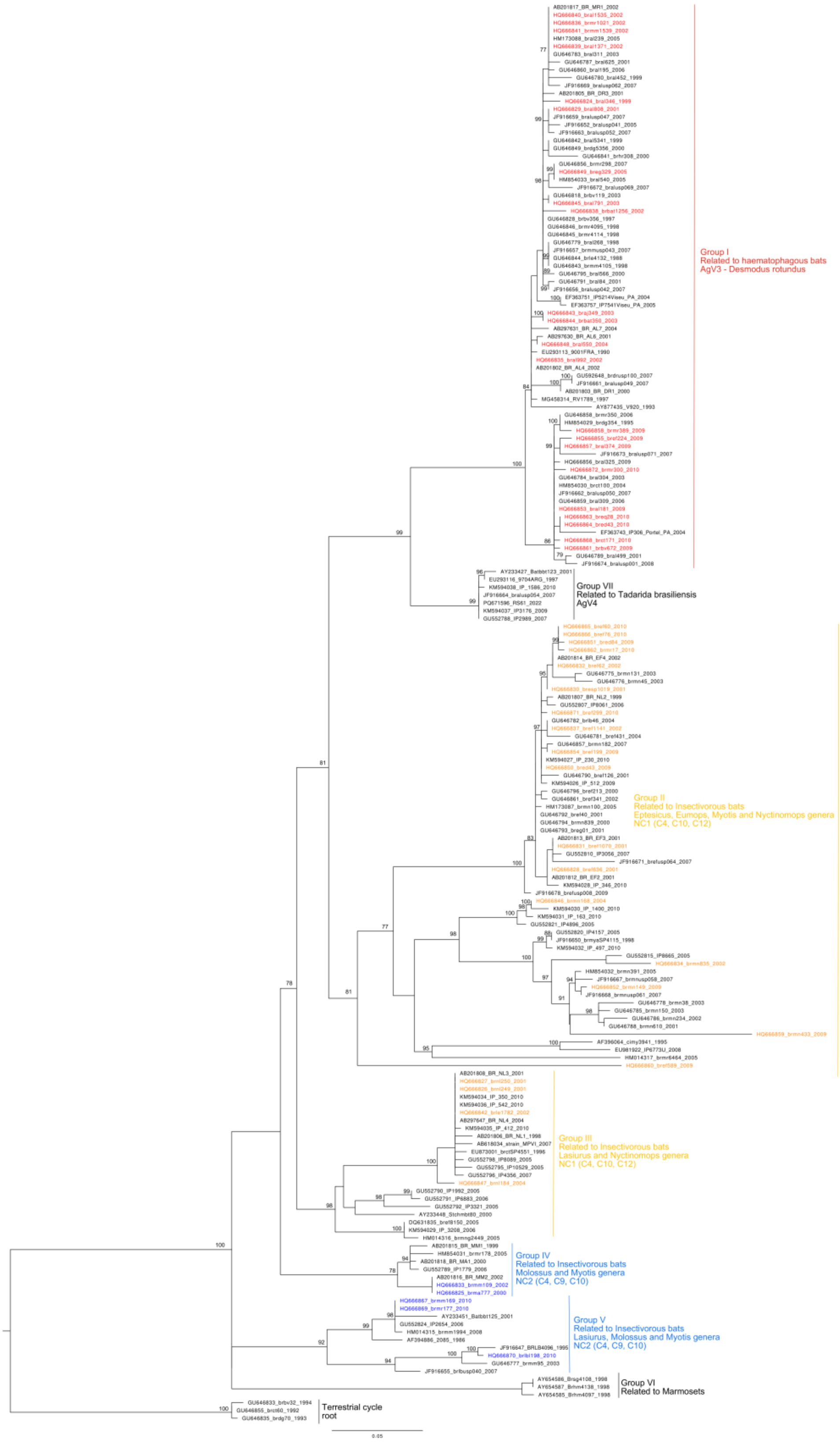

Figure S1. Phylogenetic Tree reconstructed using 320 nucleotides from nucleoprotein terminal gene using IQtree software, visualized and edited using FigTree software. Samples from Brazil and other countries, available in GenBank, are market in black. The samples from this study can be observed in the tree in red color for genetic group I, in orange (groups II and III), and dark blue (groups IV and V). The groups without segregated samples from this study are shown with pink (group VI) and gray (group VII).

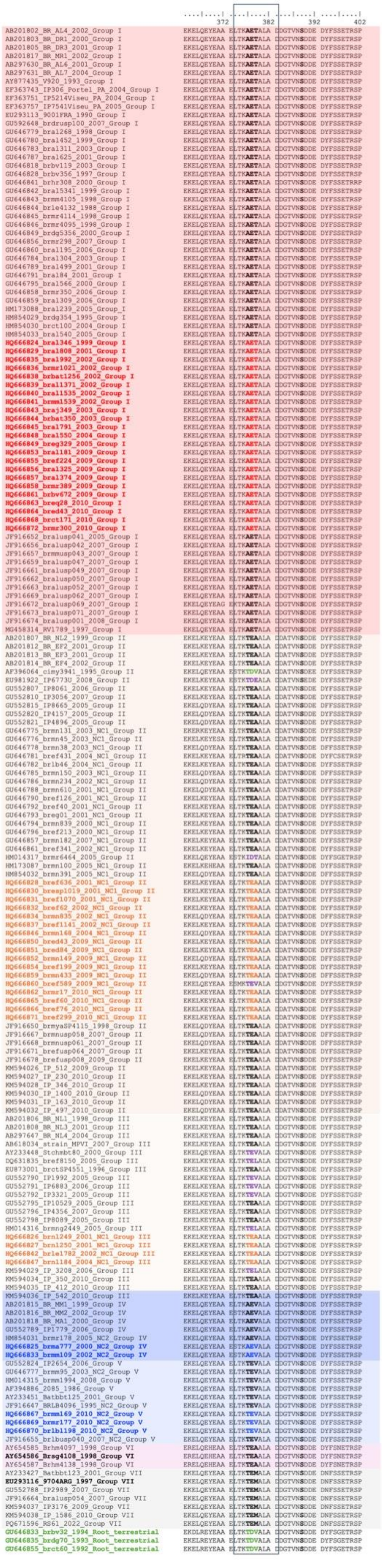

Figure S2. Partial aminoacid alignment showing the antigenic site I presents in nucleoprotein for all sequences used in phylogenetic reconstruction tree.The color match with the phylogenetic tree presented at Figure 2. For this figure were used all sequences used in the phylogenetic tree reconstruction and only the variability in antigenic site region was maintained. It is possible see the genetic signature **AET** in red (and also highlighted in transparence red) for phylogenetic group I related with hematophagous bats *D. rotundus*, **AEV** in dark blue (and also highlighted in transparence dark blue) for phylogenetic group IV related with non-hematophagous bats, **TEV** in gradient of dark blue (and also highlighted in gradient transparence dark blue) for phylogenetic group V, **TEA** in magenta/pink (and also highlighted in transparence pink) for phylogenetic group VI related with marmosets, TEM in black (and also highlighted in transparence gray) for phylogenetic group VII related with bat species *Tadarida brasiliensis* and **TDV** in green for the root group related with terrestrial cycle of transmission of RABV. For phylogenetic groups II and III (highlighted in a gradient transparence in orange) were not possible to find one genetic signature in the antigenic site, actually in these groups the variability found was diverse and can be noted in different colors, **TEA** in orange for the major antigenic site found (that was the same genetic signature found in group VI related with marmosets), purple for variations (**TEL, TDE, IDT**), dark blue for the same signature presents by groups IV and V (**TEV**), and green for the same signature presents by terrestrial cycle of transmission (**TDV**) in one sequence available at Genbank (AF396064).
